# Berberine-based strategies: novel delivery systems bring out new potential for wound healing

**DOI:** 10.1186/s13020-025-01192-0

**Published:** 2025-10-01

**Authors:** Jie Yin, Siqi Qin, Junren Chen, Napsan Wong, Cheng Peng, Dan Li

**Affiliations:** State Key Laboratory of Southwestern Chinese Medicine Resources, School of Pharmacy, Chengdu University of Traditional Chinese Medicine, Chengdu, China

**Keywords:** Berberine, Delivery system, Hydrogel, Nanofiber, Wound healing

## Abstract

Wound healing is a highly regulated biological process that restores tissue integrity following injury, while it could be disrupted in specific conditions such as diabetes mellitus (DM) and bacterial infection, leading to delayed healing and serious complications. Berberine (BBR) is a pivotal naturally occurring isoquinoline alkaloid with multiple pharmacological activities, especially anti-inflammation, anti-bacteria, anti-oxidative stress, which are beneficial for wound healing. Recently, various novel carrier materials such as hydrogel scaffold, nanofiber membrane, and nanoparticles with specific pH, temperature, glucose, ROS, near infrared light responsive and controlled release properties have been utilized as carries to improve the bioactive and bioavailability of BBR that bring out promising therapeutic effect on acute wound, diabetic wound, infected wound, and burned wound. In this review, the design, synthesis, and application of novel delivery systems loaded/based with BBR were summarized based on the characteristics of these smart materials. Remarkably, the action of BBR delivery systems on wound healing are highlighted, with the aim of promoting the development of innovative materials for drug delivery in wound healing, particularly under chronic conditions in clinic.

**Graphical Abstract:**

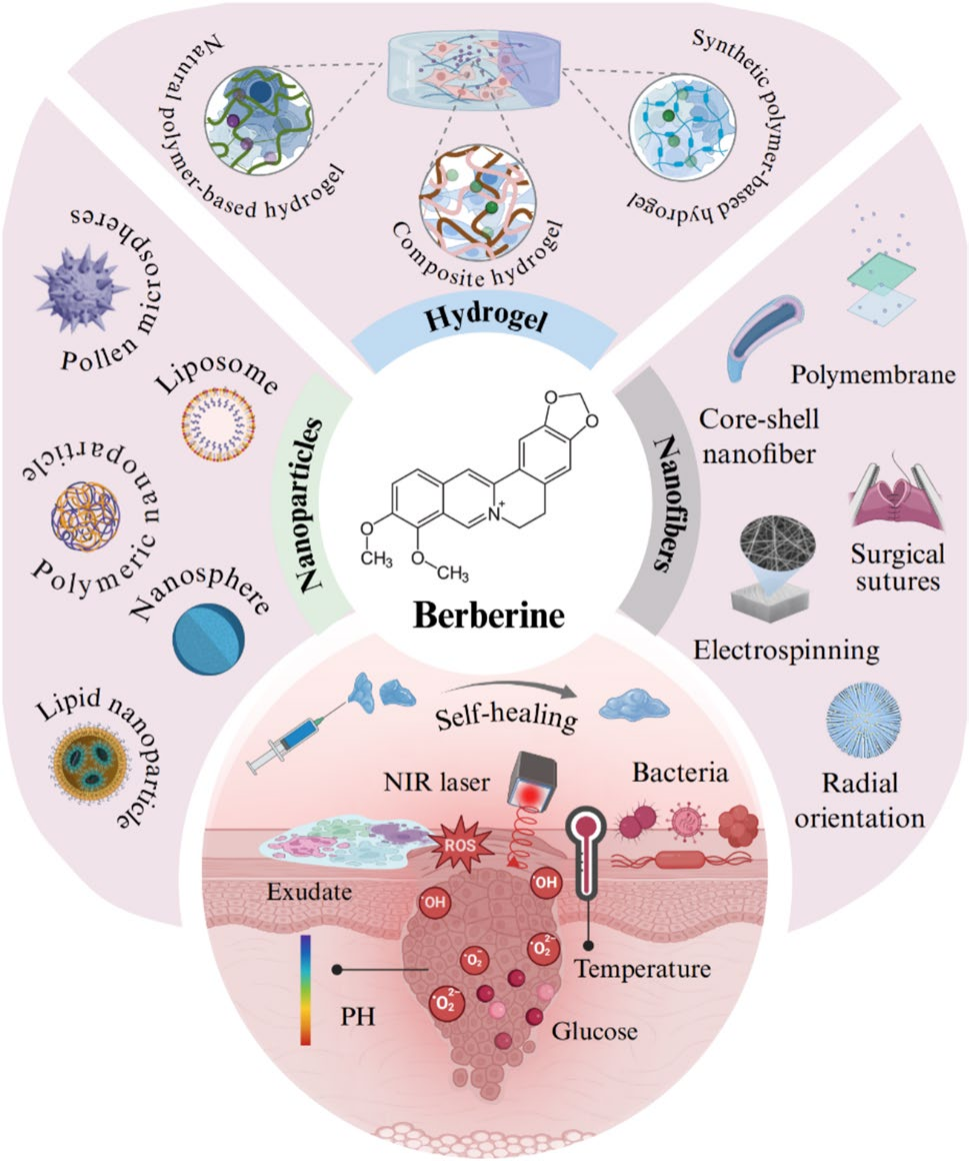

## Introduction

Wound healing is a complex, dynamic biological process that restores the integrity of damaged tissue caused by trauma, surgery, burns, or diseases such as diabetes mellitus (DM) [1]. Generally, wound healing is a gradual, overlapping process that includes hemostasis, inflammation, proliferation, and remodeling [2]. Each stage of wound healing is regulated by growth factors, inflammatory factors, and extracellular matrix (ECM) proteins to ensure the restoration of normal tissue structure. Acute wounds heal effectively through a carefully orchestrated process, whereas chronic wounds, such as those in diabetic patients, fail to pass through the normal stages of healing, leading to long-term inflammation and various complications [2–5]. Notably, it is estimated that approximately 18.6 million people with DM develop foot ulcers each year worldwide, which can further progress to soft tissue infection, gangrene, and limb loss if not treated properly [6]. Infection usually occurs when pathogenic microorganisms (bacteria, fungi, or viruses) colonize the wound site that resulting in serious inflammatory response, finally leading to delayed healing and potentially systemic complications. Additionally, infection also impairs the normal wound healing process and increases the risk of chronic wounds, sepsis, and tissue necrosis [7]. Therefore, it is crucial to find effective treatment strategies for the characteristics of different types of wounds.

BBR, a bioactive pentacyclic isoquinoline alkaloid separated from species of *Berberis*, displays outstanding anti-inflammatory and immunomodulatory activities that serve as a critical ingredient in pharmaceutical drugs and functional supplements to prevent and manage obesity and DM [8–11]. In addition, BBR possesses potent antimicrobial and antiviral effects, especially against *Staphylococcus aureus* (S. *aureus*), *Escherichia coli* (E. *coli*), influenza virus, and hepatitis B [12, 13]. Recently, ample evidence indicates that BBR displays promising therapeutic effects on various wounds, involving acute wounds (like traumatic wounds and burned wounds) and chronic wounds (such as diabetic wounds and infected wounds). However, BBR exhibits poor water solubility, low intestinal permeability, and extensive first-pass metabolism that leads to poor bioavailability. To overcome these limitations, novel carrier materials such as hydrogel, nanoparticles (NPs), liposomes, self-emulsifying drug delivery systems, and polymeric micelles have been developed, significantly enhancing its solubility, permeability, and systemic retention, thereby improving its therapeutic potential [14–18].

In this review, Literature from the past 5 years was collected from Google Scholar, PubMed, and Web of Science by keywords including BBR, wound healing, diabetic wound, infected wound, material, hydrogel, nanofiber membrane, and NPs. This review highlights the use of novel delivery systems in the management of various types of wounds, with a focus on the therapeutic potential and mechanisms of BBR and BBR delivery for surgical wounds, diabetic wounds, infected wounds, and burned wounds. In addition, future prospects of these BBR-based preparations, as well as the current insufficiencies, are discussed with a view to providing a theoretical basis for the innovation of carriers and the application of drug delivery systems for the treatment of recurrent wounds in the clinic.

## BBR-history of medicinal plants

**Fig. 1:**
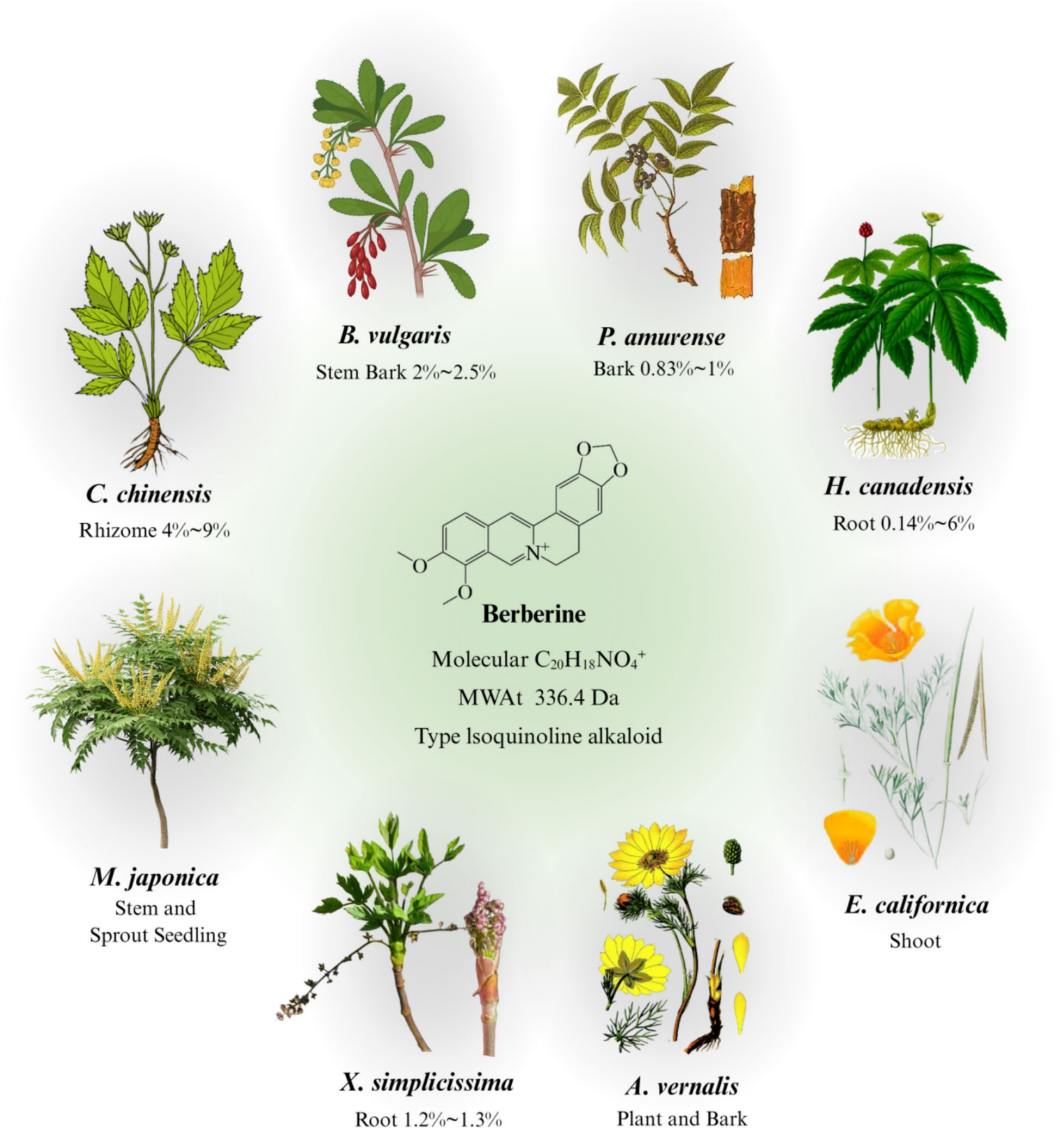
BBR chemical structure, plant sources, and respective medicinal parts and BBR content. Images sourced from the following websites: *Coptis chinensis* Franch. And *Eschscholzia californica* Cham. – Adobe Stock (https://stock.adobe.com), *Berberis vulgaris* L. – BioRender (https://www.biorender.com), *Phellodendron amurense* Rupr. – Chinese Virtual Herbarium (https://plant.ac.cn), *Hydrastis canadensis* L. – Wikipedia (https://zh.wikipedia.org), *Mahonia japonica* Thunb. – RenderHub (https://www.renderhub.com), *Xanthorhiza simplicissima* Marshall. – GBIF (https://www.gbif.org), *Adonis vernalis* L. – The Green Minder (https://thegreenminder.com)

**Fig. 2:**
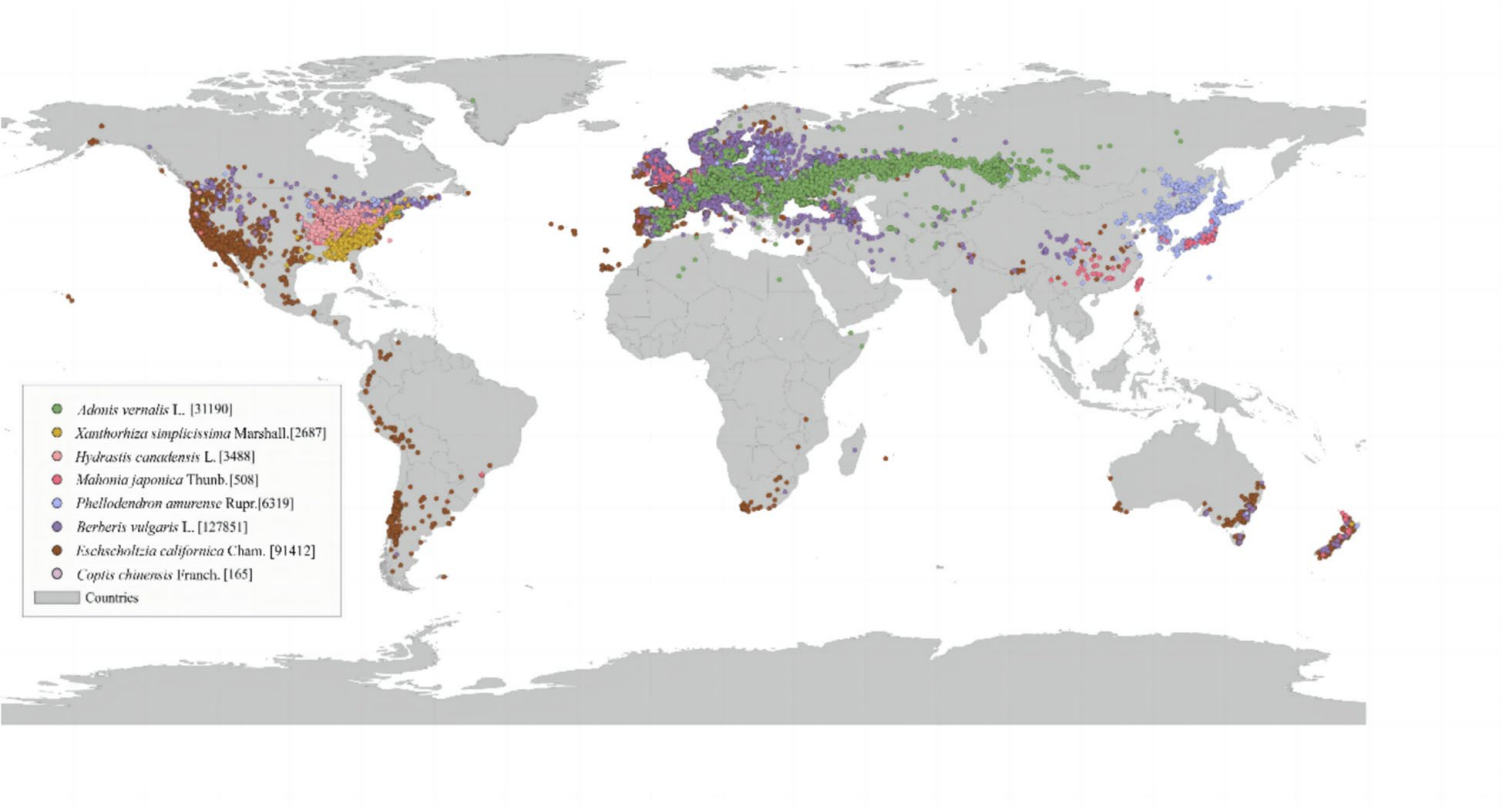
Global distribution map of BBR sourced plants and their occurrence frequency

BBR, a plant-derived alkaloid, possesses a wide range of pharmacological activities including antimicrobial [12, 13], anti-inflammatory [19], and anticancer effects [20], which is attributed to its complex isoquinoline structure (Fig. 1). BBR exists in medicinal plants particularly within the families Berberidaceae, Ranunculaceae, and Rutaceae and is extracted from the rhizomes, roots, and bark of species such as *Coptis chinensis* Franch., *Berberis vulgaris* L*.*, *Phellodendron amurense* Rupr., etc., which are now abundant and widely distributed in the Asia, Europe, and North America (version 3.40.7, https://qgis.org). (Fig. 2).

**Fig. 3:**
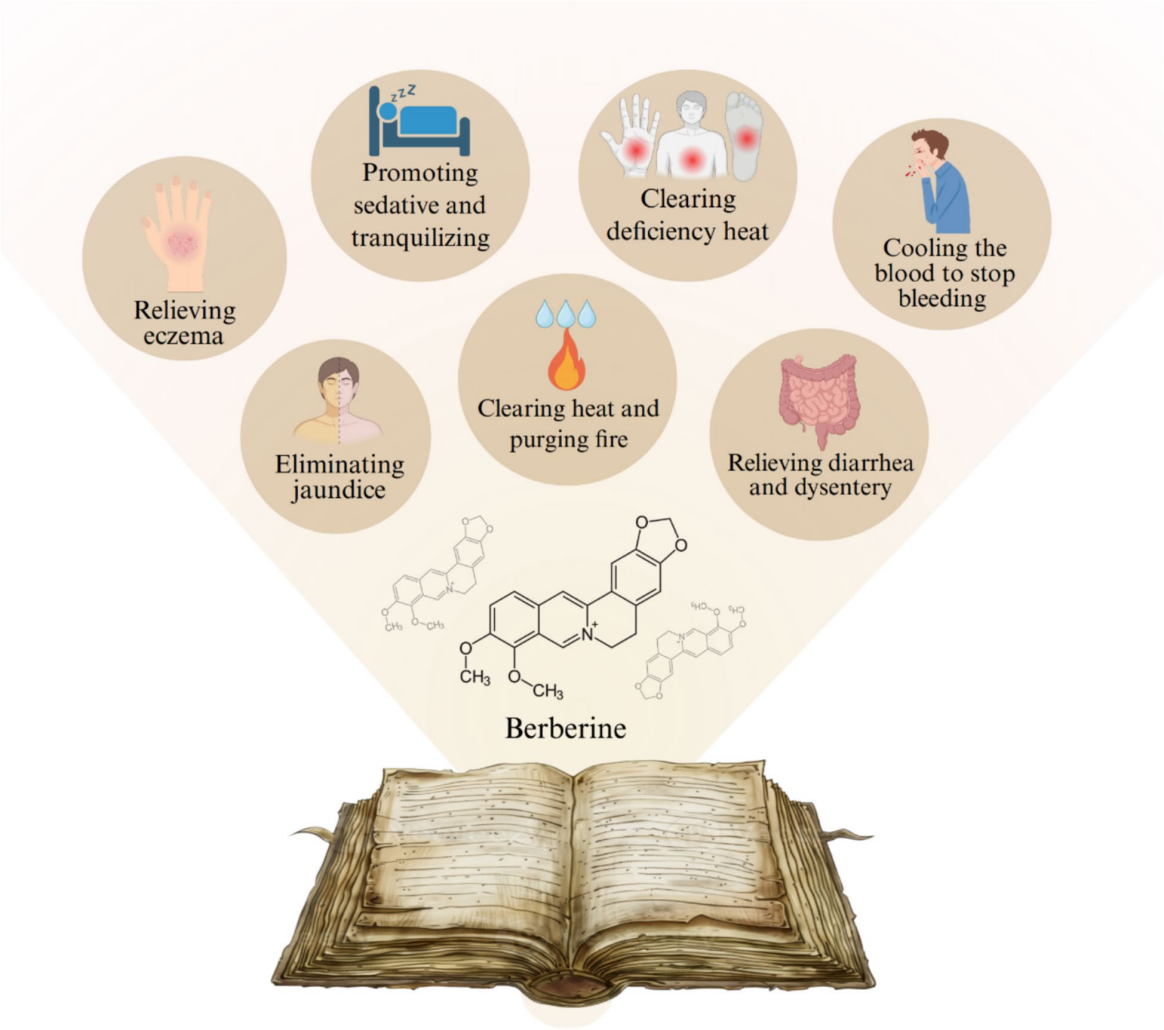
Traditional application of BBR

The pharmacological effects of BBR on wounds can be traced from historical use to modern research. As a common traditional Chinese medicinal herb, *Rhizoma coptidis* is first recorded in A.D. 200 in the book of *Shennong's Classic of Materia Medica* and described as a medicinal herb for treating skin diseases and ulcers particularly caused by “damp-heat toxic pathogens” (Fig. 3). With the primary source of BBR, *Coptis chinensis* and *Scutellaria baicalensis* are mentioned in the *Treatise on Cold-Induced Diseases* and used to treat wound infections and inflammation caused by damp-heat throughout history due to the clearing Heat and detoxifying effects. Modern scientific research has validated these ancient uses. Early 20th-century literatures have confirmed that topical treatment of BBR ameliorates the indolent ulcers [21, 22]. Afterwards, *Berberis asiatica* L. with abundant BBR is traditionally used in India and Nepal to promote for wound healing, which has been described in the *Sushruta Samhita* (1963). Later, clinical trials further validate the role of BBR in wound Healing, particularly in patients with chronic wounds and diabetic ulcers. In the past 20 years, researches have focused on the molecular mechanisms of BBR in wound healing, especially in diabetic ulcers and chronic wounds [23–25]. With the increase of studies on BBR, therapeutic effects of BBR in wounds are verified and discussed in the scientific research community.

## Novel carrier materials for BBR delivery

**Fig. 4:**
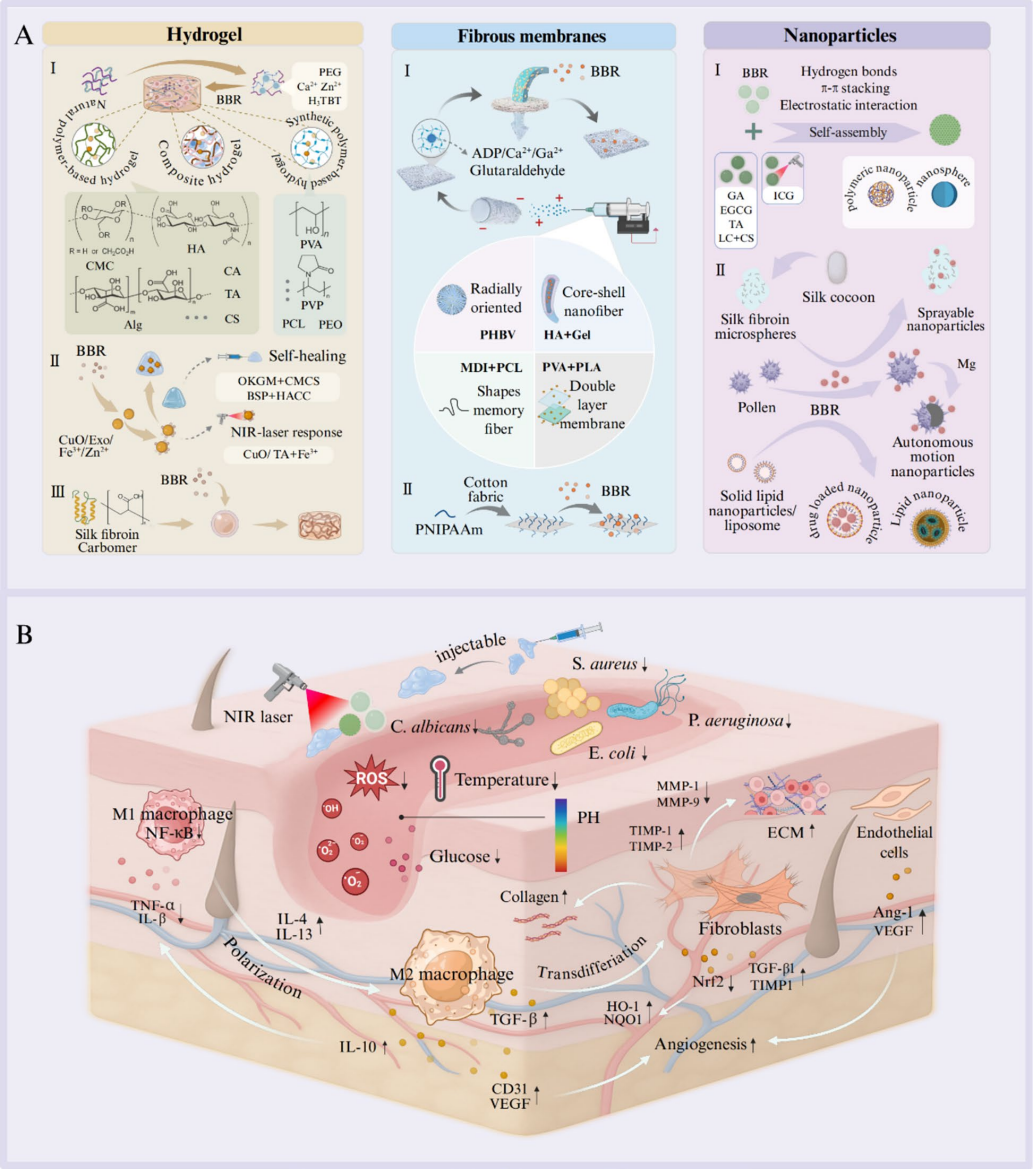
BBR-based/loaded delivery systems for wound healing. (**A**) The design of novel carrier materials for BBR delivery, including nanoparticles (e.g., liposomes, micelles, lipid nanoparticles, polymeric micelles), hydrogel (natural, synthetic, or composite), nanofibers, ECM scaffolds, and micromotors. (**B**) Biomedical applications of BBR-based/loaded delivery systems, which intelligently respond to pH, ROS, glucose, or temperature, thus regulating macrophage polarization, decrease of inflammatory state, and reduction of bacterial infection, leading to accelerate wound healing

An ideal material for wounds should meet excellent histocompatibility, good moisturizing properties, adequate physical and mechanical strength, as well as appropriate surface microstructural and biochemical characteristics to facilitate cell adhesion, proliferation, and differentiation [26, 27]. The most common synthesis of novel composite materials loaded/based with BBR are illustrated in Fig. 4A.

### Hydrogel

Hydrogel is a hydrophilic, three-dimensional, and cross-linked polymeric network. For different types of wounds, the functional hydrogel needs to possess multiple functions, including antibacterial, anti-inflammatory, antioxidant, and substance delivery to address the diverse challenges encountered during the wound healing process.

#### Natural polymer-based hydrogel

Polysaccharides used for hydrogel fabrication include cellulose, chitosan (CS), dextran, alginate (Alg), and hyaluronic acid (HA), along with their derivatives. These polysaccharides have rich chains in hydroxyl groups and/or other functional groups, such as amino and carboxyl groups, which provide versatile opportunities for preparing polymer-based hydrogel cross-linking. In addition, carboxymethyl chitosan-konjac glucomannan (CK) is a Schiff base hydrogel that is composed of carboxymethyl chitosan and oxidized konjac glucomannan, which can realize self-healing after damage and maintain drug-release properties [28]. CuO@BBR/BH hydrogel is a self-healing and injectable hydrogel prepared by dispersing CuO@BBR NPs into oxidized *B. striata* polysaccharides (OBSP) and then mixing with hydroxypropyltrimethyl ammonium chloride chitosan (HACC) solution. Interestingly, CuO@BBR NPs endow hydrogel with photothermal conversion capabilities that enable rapid heating under near-infrared light to enhance antibacterial efficacy [29]. As for now, Alg has successfully shown its credibility as a wound dressing material and will continue to serve as an ideal carrier for drug delivery [30–33]. BBR-PolyET-NC with uniform consistency, good spread ability, and compressibility is prepared by using an ionic gelation/complexation method. And the electrostatic interaction and ionic crosslinking enable CS to encapsulate the surface of the gel formed by Alg, thus enhancing the stability and controlling the release of BBR [34]. Besides, polyvinyl alcohol (PVA)/Alg hydrogel is a common hybrid hydrogel sustained-release system with outstanding mechanical properties. Using PVA/Alg as building blocks, cross-linked with calcium ions, BBR nano hydrogel (BBR-NH) possesses strong water retention and high viscosity that can maintain the wound moist and ensure a close attachment [35]. Furthermore, HA-based hydrogel regulates cellular reactions within the ECM [36, 37]. The HA/poly-L-lysine/BBR nanogel is prepared via a straightforward ionotropic gelation method, embedding BBR within a nanocarrier platform formed by electrostatic interactions between HA and poly-L-lysine, which exhibits good ECM-affinity [38]. Agarose-based hydrogel, with outstanding antimicrobial activity, is a good candidate for integration of botanicals and photothermal NPs. BBR and tannic acid (TA)-Fe(III) are loaded into agarose-based hydrogel to construct the A-T-BBR hydrogel, and near-infrared laser-activated photothermal agents TA-Fe(III) are formed by binding TA to iron. Under near-infrared irradiation (808 nm), the rise in temperature allows BBR release and physical sterilization. It is worth noting that the release of BBR under acidic conditions (pH 1 ~ 5.5) is better than under the alkaline pH value in wound [39].

Protein-based hydrogel with amino acid residues that effectively promote cell adhesion, proliferation, and differentiation and exhibit excellent pro-healing effects on various kinds of wounds [40–42]. Silk fibroin (SF) is an ideal material for wound dressing that possesses a β-sheet structure with high mechanical strength and stability. BBR contained in silk fibroin composite hydrogel (BBR-SFCH) is developed for diabetic wound healing by embedding the active substance melanin and BBR into SF-based hydrogel, which enhances cell adhesion and exudation [43]. In addition, gelatin (Gel) is a denatured water-soluble polypeptide that decomposes from the three-stranded helix of collagen into three peptide chains. Gel and sodium alginate (SA) cross-linked hydrogel exhibits excellent mechanical properties, water absorption, and biocompatibility [44, 45]. BBR/Gel/SA hydrogel is prepared by loading BBR into mTG and Ca^2+^ double-crosslinked Gel/SA hydrogel for diabetic wound healing. In addition to high swelling and porosity, such hydrogel has EDTA-induced detachment properties to prevent tear injury [46].

Interestingly, some cellulose-based hydrogels are pH-responsive and can timely release drugs to accommodate the gradual increase in pH from normal acidic range with increasing temperature to near neutral or slightly alkaline during wound healing. Bamboo parenchymal cellulose composite hydrogel loading BBR (BPCH-BBR) is prepared by in situ crosslinking of carboxylated-β-cyclodextrin, which releases drugs faster at reduced pH and increased ambient temperature through the contraction of cellulose-β-cyclodextrin network chains and the change in hydrogen bonds and electrostatic interactions [47]. Additionally, HG_sx_-BBR is prepared by adding carboxymethyl cellulose sodium salt, hydroxyethyl cellulose, and acetylated distarch phosphate to a solution containing Ga(NO_3_)_3_, followed by loading of the BBR, exhibiting high thermal stability and fluid absorption properties [48, 49]. Likewise, another report also indicates that the novel cellulose-derived HG_sx_-BBR exhibits good hydrophile range, and the penetration of BBR is effective and durable [50].

#### Synthetic polymer-based hydrogel

Polyvinyl alcohol (PVA) contains multiple hydroxyl groups that enable it to form strong intermolecular cross-links, resulting in excellent mechanical strength and elasticity. BZ hydrogel is synthesized by mixing ROS-responsive terephthalic acid with PVA, followed by the loading of the BBR@Zn-BTB NPs. BZ hydrogel with the internal structure beneficial for cell proliferation and migration can consistently release ROS-responsive drugs as the pH value increases in diabetic wounds [51]. In addition, PVA/polyvinylpyrrolidone/citric acid (PVA/PVP/CA) hydrogel is prepared by microwave irradiation, which achieves stable junctions and high oxygen permeability by obtaining tiny pore sizes through chemical bonding [52]. ZnO nano-colloids (ZnO NCs) hydrogel is prepared by loading linoleic acid and zinc sulfate into PVA/SA cross-linked hydrogel. Using ZnO NCs and BBR as raw materials, ZnO-BBR hydrogel is prepared for improving diabetic wound healing with good moisturizing and water absorption [53].

Carbomer (CBM)-based hydrogel demonstrates great promise as a medical dressing due to remarkable biocompatibility, hydrophilicity, and adhesion properties [54]. The CBM-based composite BSP/BBR hydrogel is prepared by physically loading *Bletilla striata* polysaccharide (BSP) and BBR for diabetic wounds. BSP/BBR hydrogel displays the most stable drug release ability, suitable viscoelastic solid behavior, and water retention when the mass proportion of BSP reaches 2% [55]. In addition, poloxamer is an amphiphilic copolymer composed of hydrophilic polypropylene oxide (PPO) and hydrophobic polyethylene oxide (PEO) blocks. When the temperature changes, poloxamer exhibits a reversible sol–gel transition and forms a network structure [56]. BBR-Lip gel is prepared by incorporating BBR-encapsulated liposomes into poloxamer displays a protective effect on infected wounds. Remarkably, the high cholesterol concentration in liposomes could bind to bacterial protein toxins, contributing to the potent anti-bacterial effect [57].

Encouragingly, some novel smart hydrogel-based drug delivery systems applied in cancer and spinal cord injury therapy might provide promising strategies for wound healing. A novel type of monitoring hydrogel dressing is an effective targeted and imaging agent for tumor tissue treatment by converting temperature stimulation into a “turn-on” monochromatic signal [58]. Such monitoring hydrogel dressings that respond to temperature may serve as real-time monitors of wound status, signaling infection or inflammation by turning on a detectable signal. Another innovative conductive hydrogel dressing shows good ionic conductivity and adjustable release characteristics by enhancing transdermal effect [59, 60]. These conductive hydrogels may mimic the electrical conductivity of skin and support electrical stimulation therapy, thus promoting angiogenesis and re-epithelialization. In addition, developing natural nano-carriers derived from utilizes small extracellular vesicles (sEVs) is an integrated system of drug delivery, which demonstrates excellent transport ability and crosses the blood–brain barrier [59]. Therefore, by loading sEVs, such as those derived from stem cells or immune cells, into hydrogel systems, bioactive molecules like miRNAs and growth factors could be delivered into wound site to modulate immune response and promote wound healing.

### Nanofiber membrane

Nanofiber membranes (NFMs) exist in the form of nonwoven ultrafine fibers that imitate the structure of natural ECM and provide a strong scaffold for cell proliferation and migration, relying on the characteristic of randomly arranged fibers [61–63].

Electrospinning has become a hotspot for manufacturing NFMs wound dressings due to their excellent porosity and air permeability [64–66]. As ideal electrospinning materials, the natural polymers, including cellulose, proteins, and HA [67–69], exhibit moderate molecular conductivity, viscosity, and surface tension. Cellulose acetate (CA) is the acetate ester of cellulose with good mechanical strength, solubility, and electrical conductivity. BBR-loaded CA and HA electrospun fibers (CA/HA/BBR) have good biocompatibility, nontoxicity, and ECM affinity, exhibiting outstanding effects on infected wounds [70]. Gel, as a partially hydrolyzed product of type I collagen, is widely used in electrospun wound dressings. A diabetic foot ulcer specific wound dressing (CA/G/BBR) is prepared by CA, Gel, and BBR. After electrospinning, the cross-linking glutaraldehyde steam prevents the dissolution of Gel in the biological environment. This nanofiber dressing could absorb the exudate in the wound bed while significantly promoting cell proliferation [71, 72]. Additionally, the biocompatible CZ-BBR NFMs are developed by co-electrospinning of hydrophilic collagen and hydrophobic zein, with BBR infiltration is a promising wound dressing. Based on ECM similarity, CZ-BBR exhibits outstanding cell adhesion, and its surface wettability and fiber stiffness increase with the decrease of zein [73].

The synthetic polymers endow wound dressing with stronger mechanical properties, including strength, toughness, elasticity, and non-degradability. Polycaprolactone (PCL) is rubbery and usually possesses excellent toughness, outstanding biocompatibility, and biodegradability. BBR-NFMs are prepared by mixing the high-concentration BBR solution with PCL for electrospinning with excellent breathability and moisture absorption. The load of high-concentration BBR endows it with high extensibility and excellent antibacterial properties [74]. PVA and PEO, with strong hydrogen bonds and entanglement, improve the electrospinning of worm-like structures of SA. Using CaCl_2_ as a crosslinking agent, SA/PVA/PEO/BBR electrospun microfiber membranes with ECM-like structure that have high moisture absorption, good water solubility resistance, high mechanical strength, and excellent comprehensive performance, are suitable for wound dressing application [75]. In the same way, with PEO placed in the high molecular weight and high viscosity CS chains to prevent repulsion of similar charges and disruption, the electrospinning CS/PEO/BBR constructs scaffolds with appropriate physical and mechanical structures that provide a foundation for specific morphology, adhesion, and the generation of new tissue [76]. In addition, PHBV-BBR nanofibers are formed by embedding BBR to radially oriented Poly(3-hydroxybutyrateco-3-hydroxyvalerate) (PHBV) with a modified electrospinning strategy. Interestingly, PHBV-BBR could induce rapid migration of cells from the periphery to the center along radially oriented nanofibers, and it also possesses improved surface hydrophilicity and mechanical properties [77]. The novel core–shell nanofibers (NFs) BBR-Ar-NFs, composed of the Gel-poly (vinyl alcohol) in the shell layer and the HA-poly (vinyl alcohol) in the core layer, are prepared by electrospinning. In the wound bed, BBR-Ar-NFs constantly release arginine and BBR in situ due to abundant hydrolase enzymes and then promote cell anchoring, adhesion, and proliferation [78]. The BBR/BSP/CuO dressing is made of hydrophilic inner nanofibrous layer and hydrophobic outer layer formed by PVA and polylactic acid microspheres (MPs), which is designed to mimic the epidermal and dermal structures of the wound. Such carrier could reduce the risk of bacterial infection while adapting to the internal environment of the wound [79].

The unique properties of electrospun nanofibers allow them to be made into other forms, such as sutures, dermal substitutes, and engineered skin tissues for wound healing. The incorporation of electronics into nanofiber scaffolds could realize the precise release of multiple factors at different stages, meeting the demands of different stages of wound healing [80]. Combining with electrical stimulation [81], mechanical stress [82], and pulsed magnetic field [83] could further enhance the pro-healing effect of electrospun nanofibers on wounds. For example, depositing Ag and Zn on nanofiber dressings could provide an electric field to direct cell migration and improve wound healing [84]. Poly(N-isopropylacrylamide) (PNIPAAm) is a typical temperature-sensitive polymer. By the grafting method, PNIPAAm-based BBR-hydrogel-grafted fabric (HGFs) is prepared by loading BBR NPs onto HGFs. HGFs are created by grafting thermo-sensitive PNIPAAm onto cotton fabric. It undergoes reversible swelling-contraction behavior in response to temperature changes, thereby achieving controlled BBR release for infected wound healing [85].

### Nanoparticles

Synthetic nanocarriers, including NPs, nanospheres, liposomes, and others, can be used to improve the spatial and temporal distribution of therapeutic agents in the body, thereby reducing side effects and/or enhancing therapeutic efficacy. In addition, co-assembly technology is used to generate nanocomposite particles without using carriers. Previous studies have shown that assembling BBR and other small molecules can greatly improve the antibacterial effect of BBR by specifically targeting bacteria, which is even better than some first-line antibiotics [86–88]. The carrier-free binary gallic acid (GA)-BBR NPs are self-assembled from GA and BBR through electrostatic interaction, π-π stacking, and hydrophobic interaction, which are able to continuously release BBR to about 48% in 24 h, so that the BBR monomer can reach equilibrium within 8 h to accelerate healing of *methicillin-resistant Staphylococcus aureus* (MRSA)-infected wounds [89]. Likewise, BBR-(-)-epigallocatechin-3-gallate NPs (BBR-EGCG NPs) are prepared by co-assembling BBR with EGCG through π-π interactions. With the assistance of EGCG, BBR-EGCG NPs increase the contact area between BBR and bacteria, thus improving the antibacterial effect [90]. Similarly, BBR-TA NPs are formed by co-assembling BBR and TA via hydrogen bonds and π-π stacking interactions. With a uniform size and polydispersity, the spherical NPs provide a promising nano-antibacterial agent for multi-drug resistant bacterial infections [86]. Besides, with lecithin (LC) and CS self-assembled through electrostatic and hydrophobic interactions to encapsulate BBR in the hydrophobic core and isopropyl myristate to enhance the hydrophobic core to further improve the drug encapsulation efficiency, BBR-LC-CS-NPs exhibit a sustained drug release and are physically stable for this type 2 DM [91]. Interestingly, photothermal BBR/indocyanine green NPs (BBR/IGC NPs) are self-assembled through electrostatic interaction and π-π stacking. By adding the photosensitizer ICG, BBR/IGC NPs induce NIR-responsively bacteria death directly during NIR laser irradiation, while elevated temperature triggers sustained BBR release [92].

Using another hot high-pressure homogenization technology, the solid lipid NPs delivering BBR (SLNs-BBR) containing compritol liposome has been established. With optimized drug loading and encapsulation efficiency, SLNs-BBR targets deep wounds through skin penetration and releases BBR continuously [93]. Recently, many technical challenges have remained in developing active agents that target interactions with smart delivery to the wound. For instance, dopamine-coated BBR NPs (PDA@BBR NPs) are a ROS-responsive delivery system that alleviates colonic ulcer through targeting and reducing ROS in injured sites [94]. Silver-containing NPs (AgNPs) are the most popular carrier material in dentistry with potent inhibitory properties against bacteria, fungi, and enveloped viruses among metallic NPs [95].

### Others

The shape memory BBR-incorporated polyurethane (SM-BBR-PU) fibers with good biocompatibility and non-toxicity are prepared by a simple one-step wet spinning method. This elongated SM-BBR-PU fibers suture could be easily applied to the wound while gradually restoring the length and shrinking the wound under the body temperature, due to the good thermal sensitivity and shape memory properties [96]. BBR-SF microspheres (BBR@MPs) are developed from SF using the polyethylene glycol emulsion precipitation method with good mechanical properties. Interestingly, the BBR@MP aqueous suspension has a very low viscosity that allows it to be prepared as a spray material, which could adhere stably to the ECM [97]. BBR/calcium dual-crosslinking hemostatic films (BBR-HFs) are established as a bio-composite that displays excellent physical properties, including high surface roughness, hydrophilicity, oxygen permeability, and especially good cell adhesion [98]. Additionally, by an asymmetric sputtering method, a Chinese herbal pollen-derived micromotor MG-SECS-BBR is prepared to treat gastric ulcers by sputtering a Mg layer on one side of outer membrane capsule of sunflower pollen grain exine capsules (SECs) and then loaded with BBR. Benefiting from voluntary movement and a unique spiny structure, it can move actively in the stomach and adhere to surrounding tissues [99]. A novel S8 + BBR film-forming polymeric solution (S8 + BBR FFPS) is constructed by combining carnitine cholesterol (S8) and BBR with hydroxypropyl methylcellulose and PVP K30 carriers. S8 + BBR FFPS has better dispersion and bio-adhesion properties when PVP K30 reaches 6%, with the lowest crystallization of film-forming agent [100].

Overall, these findings have identified the advantages of different composite materials for wounds. Different properties of composite materials, like size, shape, ductility, and mechanical performance, directly determine the characteristics of the BBR delivery system. Therefore, on the basis of those advanced research achievements, optimization of materials properties probably provides new perspectives and ideas for BBR delivery used in wound healing.

## Mechanisms of BBR-loaded/based materials in wounds

Trauma physiology has been proven to be highly complex at the cellular level, involving multiple regulatory axes and signal cascades. Different composite materials loaded/based with BBR promote wound healing by intelligently responding to wound site stimuli such as pH, temperature, and ROS to inhibit inflammatory response and bacterial infection (Fig. 4B). The characteristics, effects, and molecular mechanisms of novel BBR-based/loaded delivery systems for wound healing are summarized in Table 1.

**Table 1:** The characteristics and effects of BBR-based/loaded delivery systems on wounds

Materials	Dosage	Characteristics	Indicator	Refs.
Acute wounds				
Hydrogel				
BBR-encapsulated polyelectrolyte nanocomposite gel (BBR-PolyET-NC)	10 mg/25 mL	sustained drug release	↑: collagen, tube formation, fibroblasts and fibrocytes number ↓: macrophages, neutrophils number	[34]
Hyaluronan/poly-L-lysine/BBR nanogels (HA/poly-L-lysine/BBR)	5 mg/mg	phased release of drugs	↑: cell migration	[38]
BBR carried gelatin/sodium alginate hydrogel (BBR/Gel/SA)	0.5 mg/mL, 1.0 mg/mL, 1.5 mg/mL	peelability with EDTA	↓: S. *aureus*	[46]
Fibrous membranes				
BBR-loaded electrospun PCL nanofibrous membranes (BBR-NFMs)	0.5% (w/v)	hemostasis, water absorbency	↑: cell proliferation, platelet adhesion ↓: S. *aureus*, E. *coli*, MRSA, C. *albicans*	[74]
Hydrogel crosslinked surface-treated film loaded with BBRe (HG_sx_-BBR)	8.1% (w/w)	water absorption, BBR permeability	↑: SOD2, cell migration and proliferation ↓: ROS, iNOS, IDO1, NF-κB, MMP9, Ki67	[48, 50]
Dressings containing BBR loaded cellulose acetate/hyaluronic acid electrospun fibers (CA/HA/BBR)	1% (w/w)	injectable, toughness, fiber scaffold	↑: cell proliferation, tube formation, granulation, fibroblast, collagen, leukocyte, neutrophils ↓: E. *coli*, P. *aeruginosa*, B. *subtilis*, S. *aureus*	[70]
BBR-loaded co-electrospun nanofibrous membranes of collagen and zein (CZ-BBR)	0.6% (w/w), 1.2% (w/w)	ECM-mimicking	↑: cell adhesion, fluid retaining, collagen, thickness of the dermis ↓: E. *coli*, S. *aureus*	[73]
Bamboo parenchymal cellulose composite hydrogels loading BBR(BPCH-BBR)	0.5 mg/mL	pH-and temperature-response, in-situ delivery	↑: TGF-β, IL-10 ↓: TNF-α, IL-1β, E. *coli*, S.*aureus*	[47]
Polyurethane nanofiber membrane modified with chitosan/β-cyclodextrin/BBR (CS/β-CD/BBR)	15 mg/m^2^	oxygen permeability, fibroblasts-targeting	↑: survival and growth of HDFs, murine fibroblasts	[101]
Hyaluronic acid/gelatin coaxial nanofibers incorporated with BBR-arginine (BBR-Ar-NFs)	50 μg/cm^2^	ECM-mimicking, high load, in-situ delivery	↑: granulation, tube formation, collagen, VEGF ↓: IL-6, TNF-α, TGF-β	[78]
Nanoparticle				
BBR-loaded solid lipid nanoparticles (SLNs-BBR)	10 mg/mL	High BBR load, Phased BBR release	↑: collagen, SOD, GSH, catalase ↓: LPO, nucleated fibroblasts	[93]
Others				
BBR coated bio-composite hemostatic film (BBR-HFs)	3% (w/w), 6% (w/w)	hemostasis, oxygen permeability	↑: platelet adhesion, granulation, tube formation, thickness of the dermis ↓: S. *aureus*	[98]
Infected wounds				
Hydrogel				
Chitosan-konjac glucomannan hydrogel loaded with BBR and exosomes (CK@BBR&Exo)	0.5 mg/mL, 0.75 mg/mL	self-healing, injectable	↑: cell migration, epithelium, tube formation, collagen, CD31, α-SMA ↓: IL-6, TNF-α, E. *coli*, S. *aureus*	[28]
Hydrogel loaded with BBR liposome (BBR-Lip)	200 μg/mL	in-situ delivery, heat-sensitive, biofilm removal	↑: collagen, VEGF, CK14 ↓: F4/80, IL-Iβ, IL-6, TNF-α, S. *aureus*	[57]
Agarose-TA-Fe(III)-BBR hydrogel dressing (A-T-BBR)	1.0 mg/mL	NIR-responsive, photothermal	↑: collagen ↓: S. *aureus*, MRSA	[39]
Fibrous membranes				
Chitosan/polyethylene oxide/ BBR nanofibers (CS/PEO/BBR)	0.5–20% (w/w)	ECM simulation, liquid absorption	↑: collagen ↓: parasite	[76]
Copolymer hydrogel-grafted fabrics embedding of BBR nanosuspension (BBR-HGFs)	25.93–26.87% (w/w)	sustained BBR release, water absorption, reversible temperature sensitivity	↑: tube formation ↓: S. *aureus*, purulent organizations	[85]
Nanoparticle				
BBR and indocyanine green nanoparticles (BBR/ICG NPs)	50% (w/w)	NIR-responsive release, self-assembly, photothermal	↑: collagen ↓: neutrophils, S. *aureus*, E. *coli*	[92]
Nanoparticles of co-assembly from BBR and tannic acid (BBR-TA NPs)	BBR/TA (1:1, mol/mol)	self-assembly	↑: collagen, tube formation, CD31 ↓: neutrophils, ATP levels of S. *aureus* and MRSA	[86]
Gallic acid and BBR nanoparticles (GA-BBR NPs)	BBR/GA (1:1, mol/mol)	self-assembly, sustained release, biofilm removal	↑: granulation, collagen, TGFA, FGF-2, FGFR3, VEGF, tube formation ↓: rpsF, rplC, rplN, rplX, rpsC, rpmC, rpsH, TNF-α, IL-1β, IL-6, Timp1, Timp2, MMP-2, MMP-9, MRSA	[89]
BBR-(-)-epigallocatechin-3-gallate nanoparticles (BBR-EGCG NPs)	BBR/EGCG (2:1, mol/mol)	self-assembly,biofilm removal	↑: tube formation ↓: ATP levels of S. *aureus* and MRSA	[90]
Others				
BBR–silk fibroin microsphres (BBR@MPs)	2 mg/mL	sprayable, ECM anchoring, sustained BBR release	↑: cell migration, VEGF, capillaries ↓: TNF-α, IL-6, IL-1β, MCP-1, CD86, tube formation, collagen, MPO, CD86, S. *epidermidis*, S. *aureus*	[97]
Shape memory BBR-incorporated polyurethane fibers (SM-BBR-PU)	12.7%, 22.4% (mass concentration)	shape memory, heat sensitivity, sustained BBR release	↓: neutrophils, TNF-α, IL-1β, E. c*oli*, S. *aureus*	[96]
Diabetic wounds				
Hydrogel				
BBR nano-colloids hydrogel (BBR-BNH)	0.01 g/10 mL	moisture retention, Sustained BBR release	↑: cell proliferation and migration, Sirt1, SMA, VEGF, CD31 ↓: MMP, NF-κB, TNF-α, IL-6	[35]
BBR-loaded *Bletilla striata* Polysaccharide hydrogel (BSP/BBR)	0.25 g/mL	stable BBR release	↑: cell migration, DPPH scavenging, collagen, capillaries ↓: glucose, IL-6, MCP-1, TNF-α, E. c*oli*, S. *aureus*	[55]
Silk fibroin composite hydrogel containing BBR (BBR-SFCH)	0.5 mg/μL	high toughness	↑: cell proliferation, collagen, CD31, tube formation ↓: F4/80, S. *aureus*	[43]
Encapsulation of BBR decorated ZnO nano-colloids into injectable hydrogel (ZnO-BBR/H)	20 mg/20 mL	moisture retention	↑: VEGF, EGFR, FGFR, CD31, SMA, p-AKT/AKT, Nrf2, HO-1, NQO1, SOD ↓: TNF-α, IL-1β, IL-6, NO, LDH, MDA	[53]
Nanoparticles				
Hydrogel encapsulates BBR@Zn-BTB nanoparticles (BZ hydrogel)	20 mg/mL	high BBR load	↑: cell proliferation and migration, collagen, TIMP-1/MMP-9 ↓: ROS, TNF-α, IL-1β, IL-6, S. *aureus*, S*. epidermidis*, E. *coli*	[51]
composite hydrogel based on BSP and HACC encapsulated with CuO and BBR nanoparticles (CuO@BBR/BH)	100 μg/mL	self-healing, injectability, NIR-responsive	↑: cell migration, collagen ↓: neutrophils, M1 macrophages, ROS, TNF-α, MCP-1, IL-6, S. *aureus*, P. *aeruginosa*, E. *coli*	[29]
Fibrous membranes				
Electrospun cellulose acetate/gelatin nanofibrous wound dressing containing BBR (CA/Gel/BBR)	1% (w/w)	toughness, water vapor permeability	↑: cell proliferation and adhesion, tube formation, fibroplasia, collagen, epidermis ↓: S. *aureus*, P. *aeruginosa*	[71]
PHBV electrospun nanofibers encapsulated with BBR (PHBV-BBR)	1% (w/w), 5% (w/w)	radially oriented, ECM-mimicking	↑: collagens, thickness of the epidermis ↓: IL-6, TNF-α, S.*aureus*, E. *coli*, C.*albicans*	[77]
Bilayered EHDA Janus hybrids loaded with BSP-BBR and CuO (BBR/BSP/CuO)	0.2% (w/w)	double layered load, water absorption, Toughness	↑: cell proliferation, migration, collagen, granulation, tube formation, thickness of the epidermis ↓: IL-6, TNF-α, MCP-1, ROS, S*.aureus*, E. *coli*, P. *aeruginosa*	[79]
Nanoparticle				
BBR encapsulated lecithin-chitosan nanoparticles (BBR-LC-CS-NPs)	0.02% (w/v)	sustained BBR release	↑: granulation, fibroblast proliferation, tube formation, collagen ↓: inflammatory cells numbers	[91]
Other wounds				
BBR-loaded sporopollenin exine capsules asymmetrically sputtered Mg layer (MG-SECS-BBR)	–	pH-controlled movement, strong BBR adhesion	↑: IL-10 ↓: neutrophils, macrophages, TNF-α	[99]
Film-forming polymer solutions containing cholesterol myristate and BBR (S8 + BBR FFPS)	2 mg/mL	persistent release, adhesion	↑: cell proliferation, β-catenin, Wnt3a, c-Myc, Cyclin D1, LEF1, TGF-β, IL-10, IL-13, granulation ↓: GSK-3β, IL-6, TNF-α	[100]

(↑ represent promotion or increase, ↓ represent inhibition or decrease)

### Acute wounds

Balanced water absorption and optimal oxygen supply to the wound site are necessary to facilitate cellular processes such as collagen synthesis, fibroblast proliferation, and the resolution of inflammation. The modified polyurethane nanofibrous membrane CS/β-cyclodextrin (β-CD)/BBR is a promising fibroblast-targeted acute wound dressing, showing good oxygen permeability and proliferation-promoting effects in Human dermal fibroblasts and 3T3 mouse fibroblasts [101]. By optimizing water absorption, HG_sx_-BBR has the potential to promote acute wound healing by increasing SOD levels in NhDP cells and HaCaT cells and reducing iNOS, NF-κB, and MMP9 levels. In addition, HG_sx_-BBR can downregulate Ki67 to regulate keratinocyte proliferation and prevent scar formation [50, 98]. Likewise, BBR/Gel/SA hydrogel continuously supplies oxygen to the wound site and absorbs exudate, while improving the cell morphology and survival rate of L929 cells. When the internal structure is damaged, the hydrogel can be peeled off from the wound without tearing [46].

**Fig. 5:**
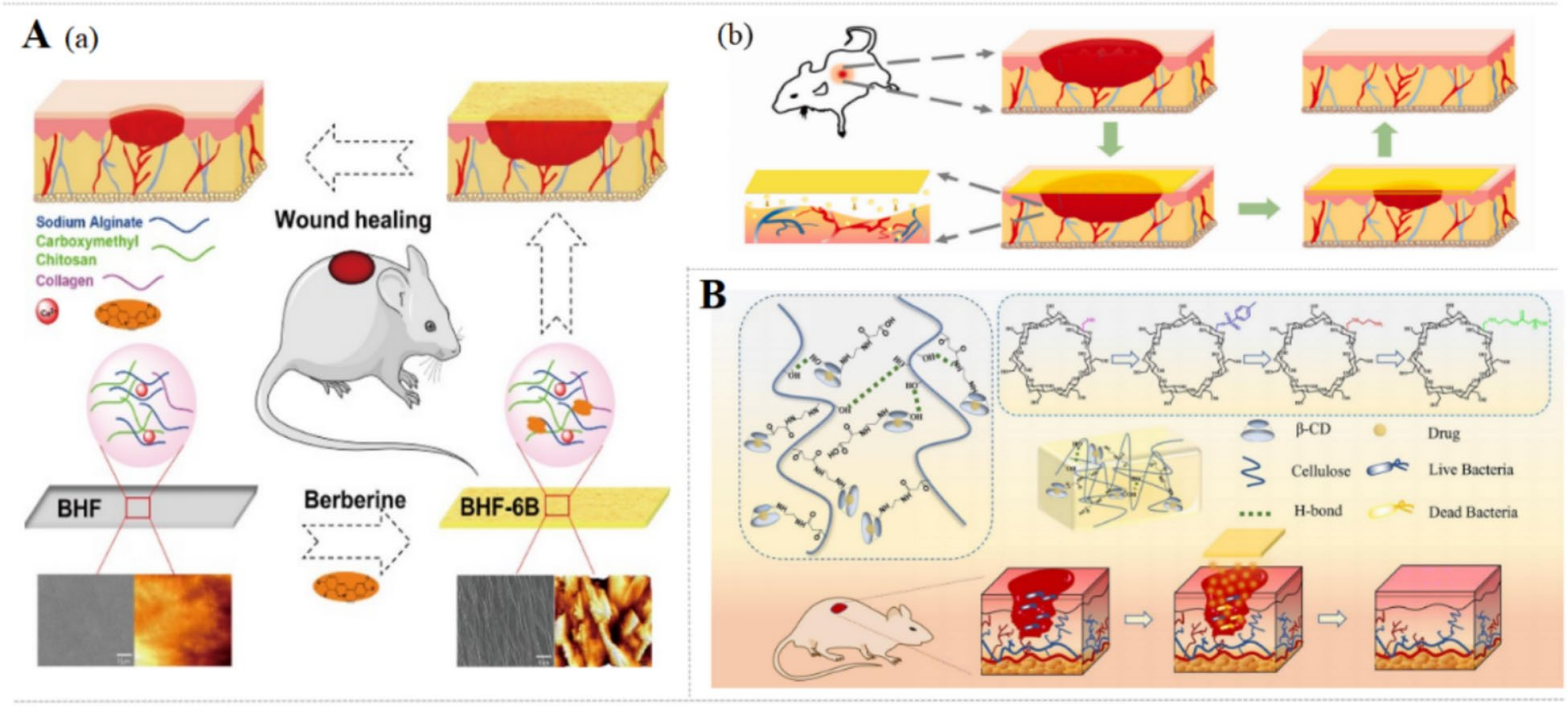
Mechanisms of BBR-loaded film and hydrogel for acute wounds. **A** The feature of the BBR-HF and BHF-6BBR (**a**), which contributes to the process of wound healing (**b**) [98]. Copyright 2022 Elsevier. **B** The design and action of multi-responsive crosslinked modified cyclodextrin-cellulose hydrogel in acute wound [47]. Copyright 2023 Elsevier

Hemostasis is the first step in the wound healing process, which involves the cessation of bleeding and the establishment of a stable clot that helps prevent further blood loss. With high water absorbency, BBR-NFMs possess great clotting ability, inducing more platelet aggregates and clusters in vitro. Besides, BBR-NFMs have the potential to promote wound healing via facilitating skin fibroblasts growth [74]. BBR-HFs (Fig. 5A) loaded with 5.221% BBR significantly improve hemostasis in rats by promoting adhesion and activation of platelets and erythrocytes. In addition, with good oxygen permeability, BBR-HFs also accelerate wound healing and skin regeneration in rats by increasing the number of hair follicles, granulation tissue, and blood vessels and promoting re-epithelialization [98].

High BBR loading ensures an adequate concentration of the active agent at the wound site, while the sustained BBR release minimizes the need for frequent dressing changes and reduces the risk of infection. BBR-PolyET-NC nanocomposite gel releases drugs continuously to promote re-epithelialization and neovascularization, finally reducing inflammatory infiltration to improve the epidermal, dermal tissue of full-thickness wounds in rats [34]. In addition, BBR-Ar-NFs could anchor cells at the wound site and sustain release of BBR to promote their adhesion and proliferation, thereby improving re-epithelialization, granulation tissue growth, and collagen production. Meanwhile, BBR-Ar-NFs enhance the expression of VEGF and TGF-β in mice, manifested by up-regulating IL-6 and TNF-α in the initial stage of wound healing and down-regulating their expression in the later stage [78]. Similarly, SLNs-BBR has a long-term effect of reducing inflammation, increasing re-epithelialization, and promoting wound healing without scar formation in mice. Besides, SLNs-BBR also improves oxidative stress in vivo by decreasing LPO and increasing SOD and GSH [102].

Phased release allows for an initial burst of the drug to rapidly address infection or inflammation, followed by a slower, continuous release to maintain therapeutic levels and promote long-term healing. With excellent in situ burst release performance, HA/poly-L-lysine/BBR nanogel platform is reported to promote migration of CCL-3T3 mouse fibroblasts [38]. In addition, controlled-release delivery system BPCH-BBR hydrogel exhibits a significant increase in BBR release (over 70%) under alkaline pH (7.6) and temperature (40℃). This dual-responsive BPCH-BBR hydrogel accelerates the healing of full-thickness skin wounds in mice via promoting re-epithelization and angiogenesis. Additionally, BPCH-BBR (Fig. 5B) shows a significant reduction of M1-associated TNF-α and IL-1β, a notable increase in the levels of M2-secreted TGF-β and IL-10, which indicates that BPCH-B can facilitate the transformation of macrophages from M1 phenotype to M2 phenotype [47].

ECM-mimicking CZ-BBR could improve adhesion and viability of fibroblasts and promote wound healing in rats through thickening of collagen fiber bundles and increasing follicle regeneration and dense connective tissues [73]. Furthermore, the fiber scaffold CA/HA/BBR improves wound healing in rats via inhibiting inflammatory cell infiltration, increasing the density of fibroblasts and collagen, and promoting granulation tissue maturation, consequently facilitating the regeneration of epidermal, blood vessel, and hair follicle [70]. Besides, the stably cross-linked PVA/PVP/CA hydrogel carrier loading BBR and chlorogenic acid has anti-inflammatory properties comparable to those of aspirin and shows great potential for acute wound therapy, which is manifested in the inhibition of the activity of albumin in vitro [52]. Hence, these carrier materials collaborate with BBR mainly promoting hemostasis, preventing bacterial infection, and providing structural support for re-epithelization, thereby accelerating the acute wounds healing.

### Infected wounds

BBR possesses powerful anti-bacterial activity and wide antibacterial spectrum through inhibiting the bacterial enzyme activity, preventing bacterial DNA replication, and effectively interfering with bacterial growth and reproduction processes. Given the potent antibacterial activity, BBR and BBR-based/loaded preparations have been developed into adjuvants for various antibiotics in recent years [103, 104]. For instance, BHFs and BBR/Gel/SA hydrogel have sustained antimicrobial properties against S. *aureus* [46, 98]. In addition, BBR-NFMs prevent wound infection through inhibiting a broad spectrum of microorganisms, such as S. *aureus*, E. *coli*, MRSA, and C. *albicans* [74]. Additionally, CZ-BBR exhibits anti-E. *coli* and anti-S. *aureus* properties [73]. BPCH-BBR shows the inhibitory effect on E. c*oli* and S. *aureus*, while the effect on Gram-positive bacteria is stronger than Gram-positive bacteria [47]. Furthermore, PVA/PVP/CA hydrogel inhibits the growth of S. *aureus* [52] (Table 1).

**Fig. 6:**
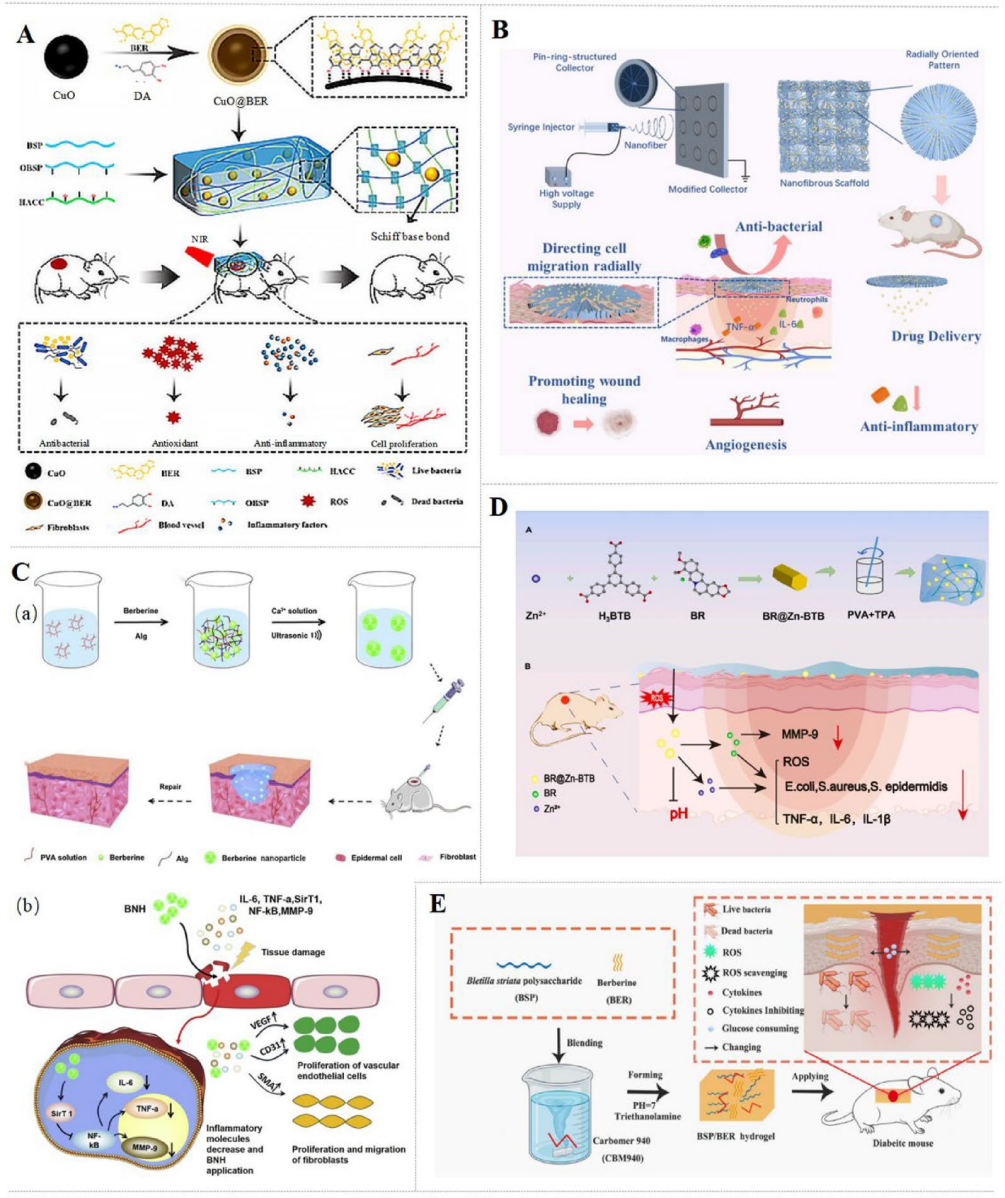
Mechanisms of BBR-based/loaded hydrogel and NPs for infected wound. **A** The fabrication of NIR responsive A-T-BBR hydrogel and its effect in the treatment of wound infections [39]. Copyright 2024 Royal Society of Chemistry. Co-assembly and antibacterial mechanism of BBR-TA NPs (**B**) [86] and BBR-EGCG NPs (**C**) [90]. Copyright 2023 MDPI and 2023 Elsevier. **D** Preparation of carrier-free BBR/IGC NPs and their effect on S.*aureus*-infection wound healing [92]. Copyright 2023 Oxford University Press. **E** Action of GA-BBR NPs in MRSA-infected wound [89]. Copyright 2023 Dove Medical Press.

CA/HA/BBR also suppresses the activities of S. *aureus*, E. *coli*, *Pseudomonas aeruginosa* (P. *aeruginosa*), and *Bacillus subtilis* (B. *subtilis*) [105]. Under near-infrared irradiation, A-T-BBR hydrogel (Fig. 6A) with good photothermal performance synergistically inhibits S. *aureus* or MRSA in wounds of mice. Moreover, A-T-BBR hydrogel ameliorates infected wounds by improving the new epidermal layers, compact collagen fiber arrangement, and dense collagen deposition [39]. Hydrogel-grafted fabrics, BBR-HGFs achieve sustainable anti-bacterial effect and significantly improve S. *aureus-*infected wound repair via reducing the infiltration of inflammatory cells and promoting granulation and capillary formation [85]. In addition, BBR-TA NPs (Fig. 6B) possess outstanding inhibitory effect on S. *aureus* and MRSA by damaging the cell membrane and inducing intracellular ATP leakage, arresting the cell cycle in S phase, and inhibiting DNA replication. Meanwhile, BBR-TA NPs improve MRSA-infected wound healing in mice through inhibiting the infiltration of inflammatory cells and promoting regeneration, re-epithelialization and angiogenesis [86]. Besides, ECM-mimicking SA/PVA/PEO/BBR microfiber membranes demonstrate exceptional single-acting anti-E. *coli* and the membrane possesses a substantial moisture content, thereby sustaining the humid milieu essential for the proliferation and potentially cellular growth of wound cells [75].

**Fig. 7:**
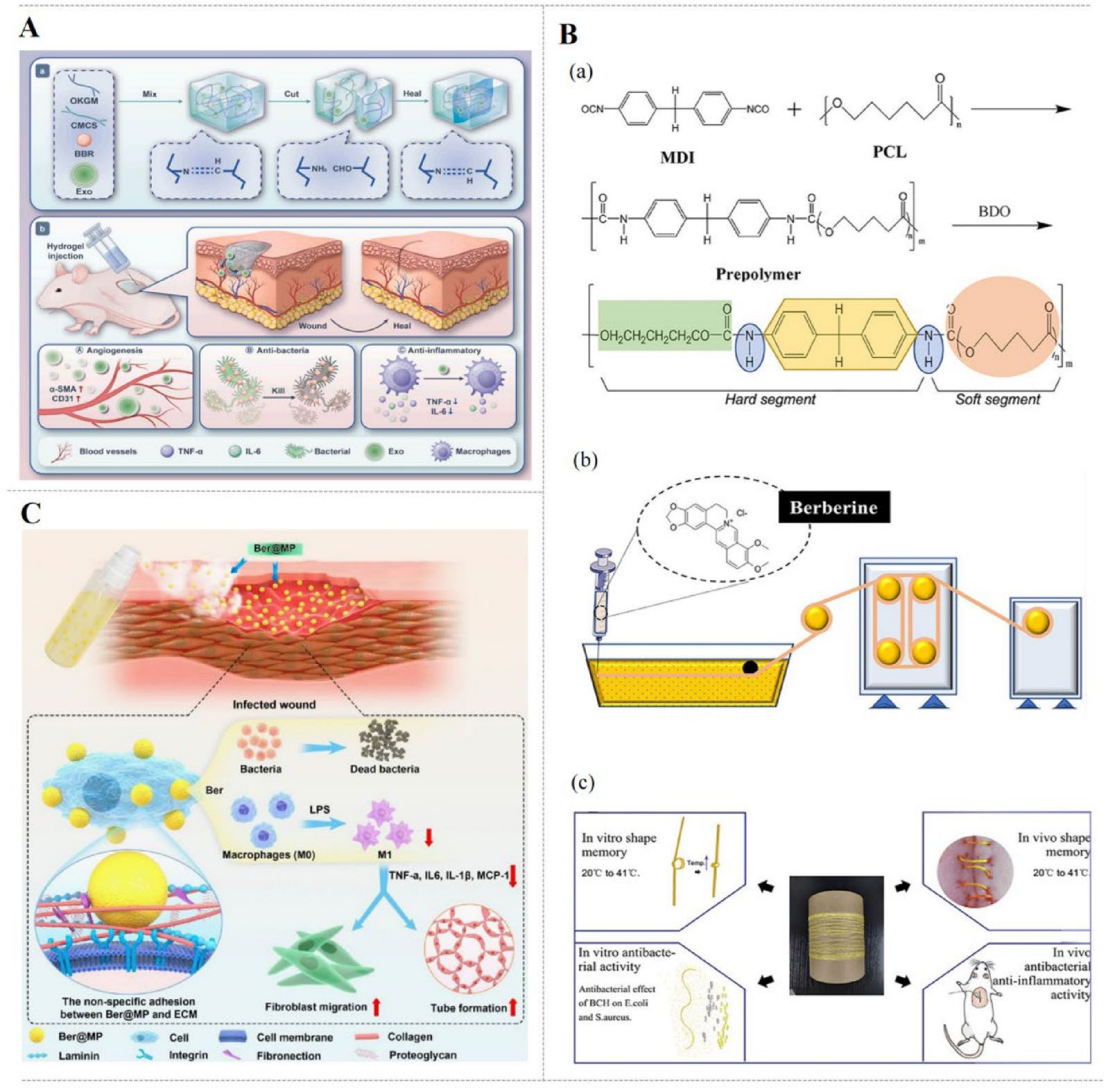
Effects of BBR-loaded preparations for infected wounds, including CK@BBR&Exo [28] (**A**), SM-BBR-PU fiber[96] (**B**), BBR-Silk Fibroin Microsphere (BBR@MP) Spray[97] (**C**). Copyright from 2023 Elsevier, 2025 Frontiers Media S.A., 2023 American Chemical Society, respectively

The injectable self-healing CK@BBR hydrogel (Fig. 7A) exhibits inhibitory on both E. *coli* and S. *aureus*, promotes endothelial cells migration, and accelerates S. *aureus*-induced wound healing in rats through inhibiting inflammation and promoting angiogenesis and collagen deposition [28]. Self-assembled BBR/IGC NPs with effective photothermal properties induce S. *aureus* and E. *coli* death directly through increasing the temperature under NIR laser irradiation. Besides, BBR/ICG NPs (Fig. 6D) ameliorate S. *aureus*-infected wounds in mice by reducing inflammatory cell infiltration, accelerating collagen fiber production and collagen deposition [92]. Interestingly, turning presentation into surgical sutures, SM-BBR-PU (Fig. 7B) inhibits the survival of E. *coli* and S. *aureus* and promotes L929 cell viability. By intramuscular implantation, SM-BBR-PU fibrous suture down-regulates TNF-α and IL-1β and reduces the number of neutrophils at the late stages of wound healing [96].

The formation of bacterial biofilms, facilitated by the protective extracellular polymeric substances, has emerged as a primary contributor to persistent infection [106]. Novel nanocomposite BBR-EGCG NPs (Fig. 6C) inhibit S. *aureus* and MRSA biofilms through promoting intracellular ATP leakage. Furthermore, BBR-EGCG NPs facilitate MRSA-induced wound healing in mice by mitigating inflammation and increasing hair follicles, sebaceous glands, and blood vessels [90]. Likewise, carrier-free binary GA-BBR NPs (Fig. 6E) eradicate MRSA biofilm by blocking the bacterial translation machinery by down-regulating the mRNA expression of rpsF, rplC, rplN, rplX, rpsC, rpmC and rpsH. Meanwhile, GA-BBR NPs accelerate MRSA-infected wound healing in rats through down-regulating TNF-α, IL-1β, IL-6, MMP-2 and MMP-9 [89]. In situ gelling hydrogel BBR-Lip increases the eradication rate of S. *aureus* biofilms, which is due to BBR-Lip targeting biofilms and sequestering bacterial toxins and cholesterol binding to protein toxins. BH-Lip also promotes S. *aureus*-induced wound recovery via up-regulating the expression of CK14 and VEGF and down-regulating the level of F4/80, IL-β, IL-6, and TNF-α [57].

In addition, there are several agents for other types of infected wounds. ECM-anchoring BBR-SF microspheres (BBR@MPs) (Fig. 7C) are firmly anchored to L929 cells and show long-lasting antibacterial activity against *Staphylococcus epidermidis* (S. *epidermidis*) and S. *aureus*. Moreover, BBR@MPs down-regulate the expression of TNF-α, IL-6, IL-1β, MCP-1 and CD86, while up-regulate that of VEGF in LPS-induced RAW264.7 cells and EA.hy926 cells. Furthermore, BBR@MPs alleviate S. *aureus*-infected wounds in mice via enhancing collagen synthesis and tube formation [97]. Skin leishmaniasis is the ulcers caused by tropical Leishmania parasites. CS/PEO/BBR nanofibers inhibit parasites to prevent infection and accelerate ulcers healing by inhibiting the inflammation and promoting the repair and construction of epidermis, dermis and collagen [76]. Therefore, BBR based/loaded delivery system improves the anti-bacterial activity of BBR on infected wounds via targeting bacterial biofilms, inducing the bacteria death directly under NIR laser irradiation, isolating protein toxins, and promoting ATP leakage, thus treating infected wounds.

### Diabetic wounds

Wound healing in DM is associated with persistent inflammation, excessive oxidative damage, insufficient angiogenesis, low collagen secretion, and abnormal tissue remodeling [107, 108]. High levels of advanced glycation end products in DM cause chronic and systemic inflammation, and this inflammation impairs collagen synthesis [109, 110]. Furthermore, oxidative stress elevation leads to dysfunctional collagen synthesis and stimulates MMP production, which induces excessive ECM remodeling [111, 112]. Therefore, advanced material that provide controlled release of therapeutic agents, along with proper pressure management, are essential for reducing inflammation, enhancing oxygenation, and supporting tissue regeneration [113].

Increasing evidences demonstrated that BBR displays outstanding effect on diabetic wound healing. BBR up-regulates the expression of Ki67 and TrxR1, inhibits the expression of p-JNK, thereby suppressing apoptosis and promoting proliferation of HaCaT cells under HG condition. In addition, BBR significantly increases GSH, SOD and T-AOC and reduces ROS and MDA in diabetic rats and promotes healing by down-regulating MMP9, TGF-β1 and MMP1 and up-regulating TIMP1 in diabetic rats [24]. Topical treatment of BBR promotes wound restoration in diabetic rats through decreasing IL-17, IL-6, IL-1β, Cxcl1, Ccl2, MMP9, and MMP3. Meanwhile, BBR facilitates ECM synthesis, angiogenesis and collagen synthesis and inhibits oxidative damage and apoptosis by up-regulating the expressions of TGF-β1, TIMP1, CD31, T-AOC, and down-regulating cleaved caspase-3 [23].

**Fig. 8:**
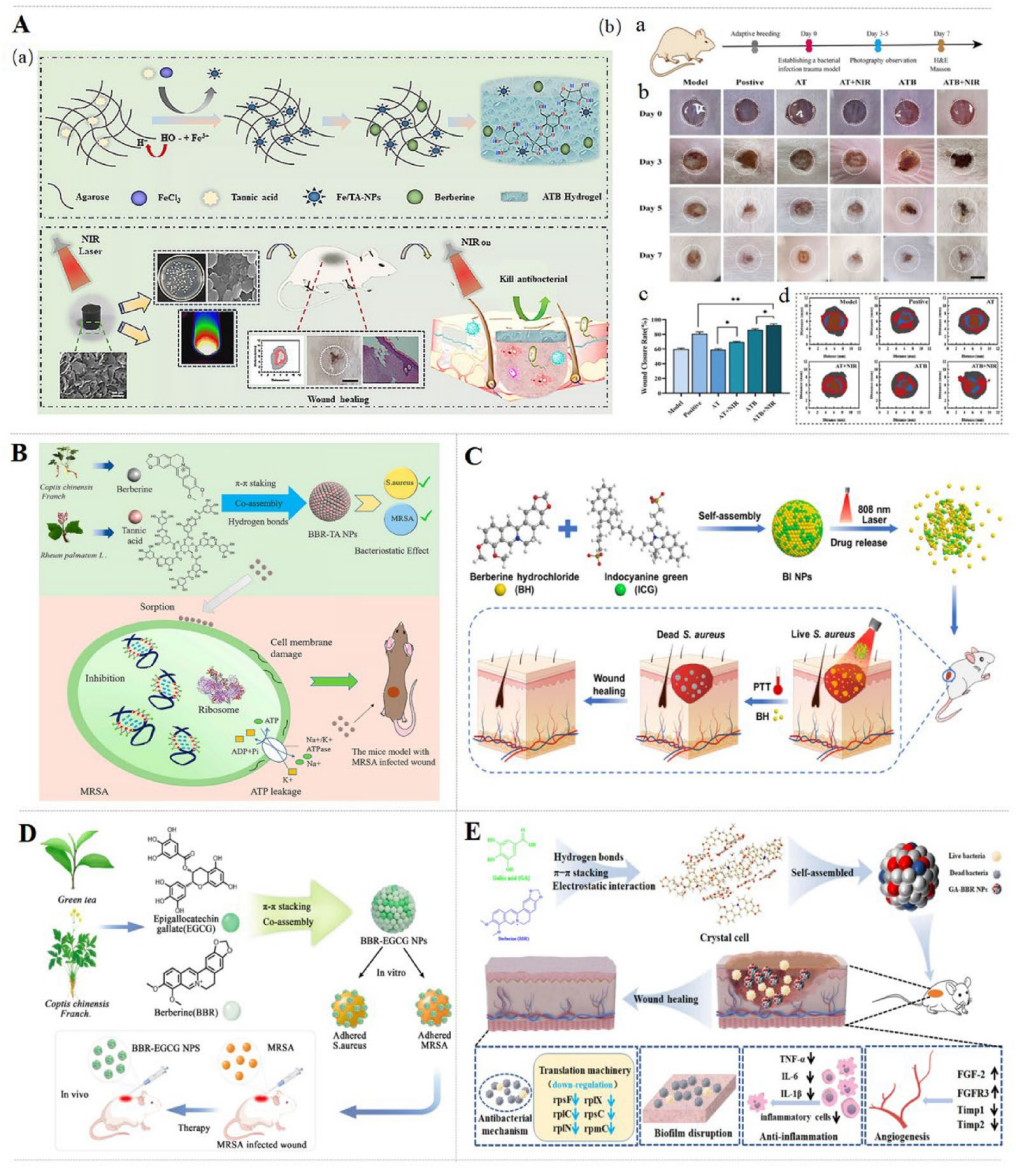
The preparations and effects of BBR-loaded delivery systems for diabetic wounds. **A** Synthetic process of an NIR-responsive photothermal CuO@BBR/BH hydrogel and its application in diabetic and infected wounds [29]. Copyright 2024 American Chemical Society. **B** The preparation process of BBR-loaded PHBV radially oriented nanofiber patches and their mechanisms for promoting diabetic wound healing [77]. Copyright 2024 Oxford University Press. **C** Preparation process of BBR-BNH and its mechanisms in diabetic wound healing [35]. **D** The synergistic effects of ROS-responsive hydrogel coupled with BBR@Zn-BTB in diabetic wound [51]. Copyright 2023 Elsevier. Copyright 2019 Elsevier. **E** The simple preparation method for BSP/BBR hydrogel and its mechanism for promoting diabetic wound healing [55]. Copyright 2023 MDPI

Delivery systems designed to target dysregulated inflammation demonstrate potent effect in the treatment of diabetic wounds. The injectable CuO@BBR/BH hydrogel (Fig. 8A) reduces the levels of IL-6, TNF-α and ROS in LPS-induced macrophages and exhibits potent antibacterial effect against S. *aureus*, E. *coli*, and P. *aeruginosa*, especially under near-infrared irradiation. Besides, CuO@BBR/BH hydrogel accelerates S. *aureus.* infected-wound healing via inhibiting inflammation, promoting re-epithelialization, collagen deposition, and angiogenesis in diabetic mice [29]. The radially oriented PHBV nanofiber dressings PHBV-BBR (Fig. 8B) reduce IL-6 and TNF-α of inflammatory macrophages and exhibit strong antibacterial activity against S*. aureus*. In addition, PHBV-BBR promote wound healing in diabetic mice by enhancing re-epithelization, blood vessel formation, collagen deposition, and hair follicle regeneration [77]. The asymmetric wettability Janus dressing BBR/BSP/CuO effectively reduces the levels of IL-6, TNF-α, MCP-1, and ROS in macrophages. The hydrophilic inner layer of the BBR/BSP/CuO promotes cell adhesion and proliferation, and the hydrophobic outer layer provides a protective barrier that prevents infections caused by pathogens. Besides, BBR/BSP/CuO suppresses inflammation and promotes epithelial regeneration and collagen deposition in a diabetic mouse model [79].

Composite materials targeting ECM deposition present a promising strategy for diabetic wound management. Angiogenesis, collagen deposition, and fibroblast migration are critical components of ECM deposition during wound healing. BBR-BNH nano hydrogel (Fig. 8C) is conducive to wound healing in diabetic rats through activating SIRT1 to increase the expression of VEGF, CD31 and SMA, as well as to inhibit the expression of NF-κB, TNF-α, and IL-6 [35]. The composite system BBR@Zn-BTB/Gel (denoted as BZ hydrogel) (Fig. 8D) promotes cell proliferation and migration of HG-induced HSF cells, while efficiently scavenging ROS. The macroporous structure of BZ hydrogel can control the release of Zn^2+^ and BBR, therefore exhibiting a broad-spectrum antibacterial effect on S. *aureus*, S. *epidermidis* and E. *coli*., and facilitates wound healing via down-regulating TIMP-1/MMP-9 ratio, TNF-α, IL-1β and IL-6, and enhancing collagen deposition in diabetic mice [51]. In addition, CA/Gel/BBR demonstrates robust microbial barrier properties, effectively inhibiting the growth of S. *aureus* and P. *aeruginosa*. With the increase of BBR concentration in the dressing, hemolysis is minimized and the proliferation of L929 fibroblasts is promoted. Additionally, CA/Gel/BBR dressing promotes wound healing in diabetic rats through improving collagen synthesis and angiogenesis as well as inhibiting epidermal hyperplasia and inflammation [71]. While BBR-LC-CS-NPs NPs achieve a good effect in accelerating diabetic wound healing by reducing inflammation, inducing vascular and fibroblast proliferation, and promoting mature collagen fiber deposition [91].

Reducing oxidative stress represents a promising direction for diabetic wound dressings. The composite hydrogel BSP/BBR enhances DDPH scavenging in H_2_O_2_-induced L929 cells and down-regulates IL-6, MCP-1 and TNF-α in macrophages. Notably, BSP/BBR hydrogel reduces the local blood glucose level in the wound area, which might be related to the activation of GLUTI. Additionally, BSP/BBR hydrogel also reduces inflammation and increases epidermis, new capillaries, and hair follicles in diabetic mice [55]. The BBR-loaded SF-melanin composite hydrogel (SFCH) with a highly porous structure is suitable for cell penetration and proliferation in H_2_O_2_-induced NIH3T3 cells. Both long-term antioxidant melanin and the slow BBR release protect wounds from pathogens. Moreover, BBR-SFCH accelerates wound healing in diabetic rats by promoting re-epithelialization, collagen deposition, skin gland and blood vessel generation [43]. BBR-modified ZnO nano-colloids hydrogel (ZnO-BBR/H) (Fig. 8E) increases the expression of EGFR and FGFR in HaCaT cells and down-regulates the expression of NO, LDH, MDA in NIH-3T3 cells, thereby enhancing cell migration and proliferation. In addition, ZnO-BBR/H accelerates wound healing in diabetic rats via up-regulating the expression of VEGF, CD31, EGFR, FGFR, α-SMA, and AKT/p-AKT and down-regulating the expression of TNF-α, IL-1β and IL-6. Besides, ZnO-BBR/H inhibits oxidative stress by up-regulating Nrf2, HO-1, NQO1, up-regulating SOD [53]. Collectively, the novel composite materials accelerate the diabetic wounds healing through enhancing the effects of BBR as reflected by the inhibition of chronic inflammation and oxidative stress and the facilitation of angiogenesis and collagen deposition.

### Other types of wounds

**Fig. 9:**
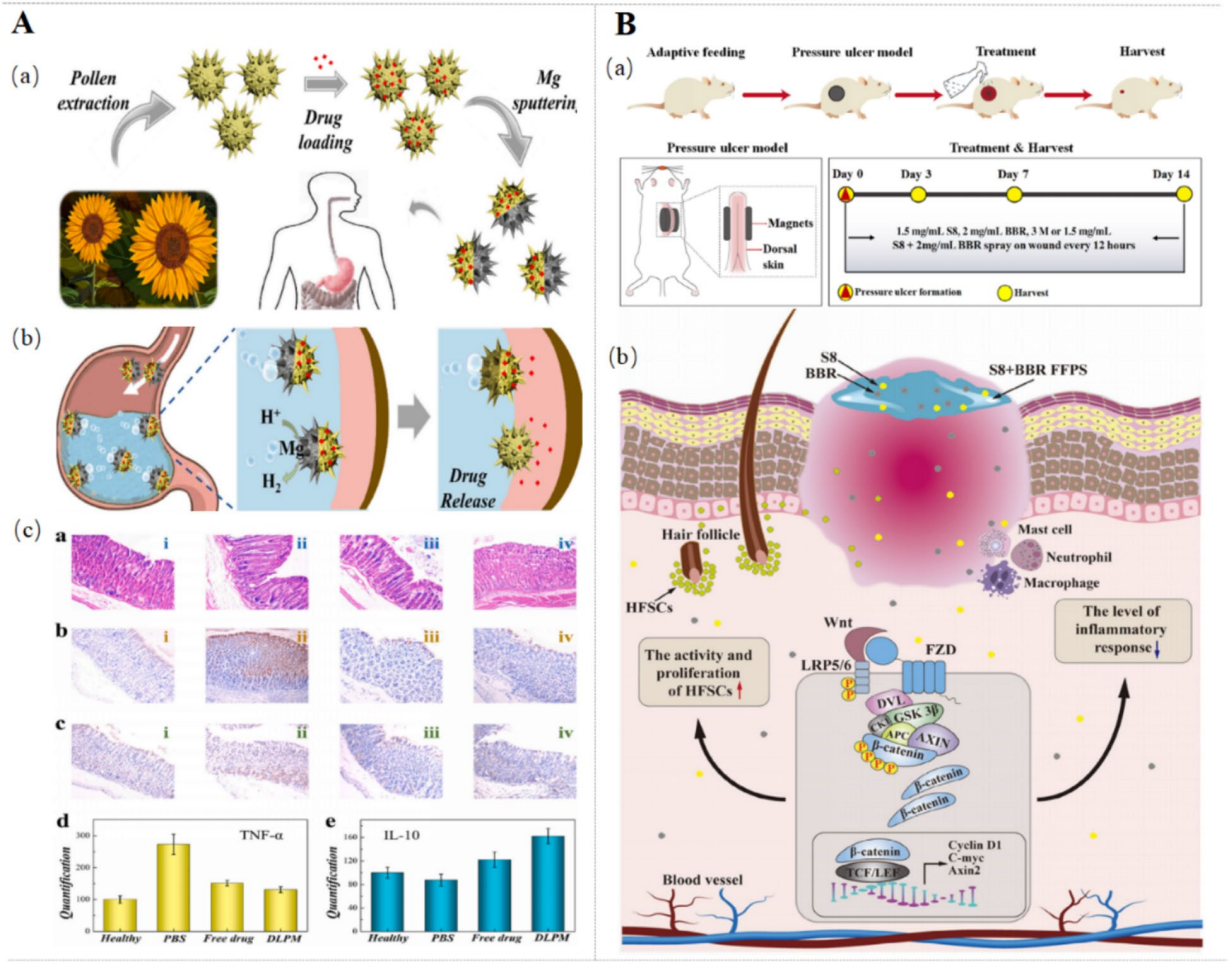
Effects of BBR-loaded delivery systems for other types of wounds. **A** Sunflower seeds loaded with BBR improve the gastric ulcers [99]. Copyright 2023 Elsevier. **B** Schematic diagram of cholesterol myristate (S8) + BBR film-forming polymeric solution in pressure ulcers [100]. Copyright 2024 The Wound Healing Society

Smart BBR-based materials also display therapeutic effects on other types of wounds, such as burns, gastric wounds, and pressure ulcers. MG-SECS-BBR micromotors (Fig. 9A), derived from Chinese herbal pollen, exhibit active gastric motility and tissue adhesion due to their spiny morphology. Mg-SECS-BBR administration enhances ulcer healing in acetic acid-induced gastric ulcer models in mice by reducing epithelial shedding, edema, and inflammatory cell infiltration, and accelerating epithelial regeneration [99]. S8 + BBR FFPS (Fig. 9B), a novel film-forming polymeric solution, demonstrates potential for pressure ulcer (PU) repair, manifested by upregulating TGF-β, IL-10 and IL-13 and downregulating IL-6 and TNF-α. S8 + BBR FFPS promotes PU wound healing via activating the Wnt/β-catenin pathway with enhanced HFSC proliferation, hair follicle regeneration, granulation tissue formation, and re-epithelialization [100]. BBR demonstrates therapeutic potential against venous leg ulcers (VLU) through modulation of the miR-21-3p/RAGB axis, which enhances vascularization and granulation tissue formation, and restores the proliferative, migratory, and tube-forming capacities of EPCs in VLU patients by downregulating RRAGB, mTOR, and p-mTOR [114]. Overall, these findings demonstrate that BBR-based/loaded delivery system facilitates the various ulcers recovery through reducing the inflammatory response and promoting the re-epithelization via modulating the Wnt/β-catenin pathway and miR-21-3p/RAGB axis.

## Conclusion and perspective

Over the past decade, BBR has garnered significant attention for its wound healing potential due to its antimicrobial, anti-inflammatory, antioxidant, and anti-diabetic properties, while its clinical application is hindered by poor aqueous solubility, low bioavailability, and rapid systemic clearance. Novel BBR-based drug delivery systems including NPs, hydrogel, microneedles, electrospun nanofibers, and bioengineered scaffolds, could enhance the therapeutic efficacy of BBR in treating different types of wounds. BBR-based delivery systems provide localized and sustained drug release, which reduce local oxidative stress and inflammation and promote the migration of endothelial cells and fibroblasts towards the wound site, thus accelerating acute wound closure. Notably, for diabetic wounds with impaired paracrine communications and chronic inflammation, hydrogel-based and nanoparticle-encapsulated BBR formulations could promote angiogenesis, fibroblast proliferation, and provide structural support and signaling cues to guide ECM remolding via regulating macrophages-driven inflammation as well as the cross-talk between macrophages, keratinocytes, and endothelial cells. Furthermore, some BBR-based composite materials, especially containing the metal ions, are particularly beneficial for infected wounds through preventing biofilm formation and combating multi-drug-resistant bacteria such as S. *aureus*, P. *aeruginosa*, and B. *subtilis*. Additionally, burn wounds, which require long-term wound management, profit from BBR-loaded electrospun nanofiber scaffolds that could mimic the ECM while providing controlled drug release to promote skin regeneration, angiogenesis, and prevent secondary infections.

Although some clinical studies have already shown that BBR blending with sesame oil ameliorates the bullous pemphigoid and nano BBR gel promotes the burn wounds healing via reducing inflammation and preventing bacterial infection [115, 116] and some patents have been authorized (Table 2, https://patentscope2.wipo.int), the clinical translation of BBR-loaded/based delivery system faces several challenges. One of the primary concerns is the stability of these novel composite materials, particularly for nanocarriers and hydrogel, which require precise physicochemical properties to ensure effective drug release. Additionally, although in vitro and preclinical animal studies have demonstrated promising therapeutic effects of novel BBR-loaded/based delivery systems, further clinical trials are necessary to validate their safety, efficacy, and long-term biocompatibility in human patients. For example, some BBR-loaded hydrogel might exhibit burst release or inconsistent drug diffusion, which might cause inadequate therapeutic effects or local toxicity. In addition, during the acute inflammatory phase of wound healing, macrophage polarization towards the M1 phenotype facilitates phagocytosis of cellular debris and bacteria at the wound site, and premature release of BBR may be detrimental to the role of these M1 macrophages [117, 118]. Interestingly, the development of stimuli-responsive BBR delivery systems that responds to wound conditions (e.g., pH-, glucose-, ROS-, or temperature-sensitive hydrogel) might optimize drug release at the wound site. However, these novel stimuli-responsive carrier materials also face considerable challenges, as the temperature, pH, and levels of glucose and ROS in diabetic or infected wounds are extremely variable, which might confuse the materials to detect and conduct accurate signals to support the timely release of BBR. These unsolved issues are the reasons that limit the clinical application of the BBR based/loaded delivery system, which may lead to the failure of regulatory approval. Though the clinical application is limited, increasing studies indicate that combination therapies incorporating BBR with growth factors, stem cells, or other natural bioactive compounds might enhance wound healing synergistically [119, 120]. Furthermore, exploring more effective carrier materials may be a strategy for the future development of new types of delivery systems for wounds. For example, microneedle drug delivery systems directly penetrate the stratum corneum to reduce drug retention [121–123], the ability of nanozymes to regulate catalytic activity and types in response to external stimuli [124–126], and the presentation in the form of ECM scaffolds [127, 128] and natural sponges [129–131].

**Table 2:** Patents of BBR based/loaded delivery system for wound healing

Patent number	Title	Preparation	Wound type	Year
ZL201811225912.3	Preparation Method and Application of Antibacterial Hemostatic Microspheres Containing berberine	Hemostatic microspheres containing BBR	Normal wound	2018
ZL201811483221.3	Hemostatic Microspheres Containing Antibacterial Component berberine and Their Preparation Method and Application	Hemostatic microspheres containing BBR	Normal wound	2018
ZL202111316731.3	Crosslinked Chitosan Microspheres and Their Application in Hemostasis and Wound Healing	Cross-linked chitosan microspheres based on polyglutamine-modified chitosan and BBR nuclear layers and heparin sodium and alginate shell layers	Normal wound	2021
ZL202410386789.2	Preparation Method of Multifunctional Nanofiber Membrane for Promoting Complex Infected Wound Healing	Nanofiber membrane loaded with BBR	Infected wound	2024

In conclusion, BBR-based/loaded novel delivery systems display great promise for the treatment of various types of wounds, including acute wounds, diabetic ulcers, infected wounds, and burns. More importantly, the integration of NPs, hydrogel, microneedles, and nanofibers has significantly improved the bioavailability, stability, and wound-healing efficacy of BBR. Therefore, optimization of these carrier materials loaded with BBR, evaluation of their safety, and in-depth exploration of their mechanisms of promoting wound healing may contribute to the improvement of smart materials for drug delivery systems in clinical treatment of refractory wounds.

## Data Availability

No datasets were generated or analysed during the current study.

## Abbreviations

Alg: Alginate
BBR: Berberine
BSP: *Bletilla* *striata* Polysaccharide
β-CD: β-Cyclodextrin
B. *subtilis*: *Bacillus subtilis*
CA: Cellulose acetate
CBM: Carbomer
C. *albicans*: *Candida albicans*
CK: Chitosan-konjac glucomannan
CS: Chitosan
PDA: Dopamine
DM: Diabetes mellitus
E. *coli*: *Escherichia coli*
ECM: Extracellular matrix
EGCG: (-)-Epigallocatechin-3-gallate
Gel: Gelatin
FFPS: Film-forming polymeric solution
GA: Gallic acid
HA: Hyaluronic acid
HACC: Hydroxypropyl trimethyl ammonium chloride CS
HGFs: Hydrogel-grafted fabrics
ICG: Indocyanine green
LC: Lecithin
MPs: Microspheres
MRSA: *methicillin-resistant Staphylococcus aureus*
NH: Nano hydrogel
NPs: Nanoparticles
NFs: Nanofibers
NFMs: Nanofiber membranes
PCL: Polycaprolactone
PEO: Polyethylene oxide
PU: Pressure ulcer
PVA: Polyvinyl alcohol
PVP: Polyvinylpyrrolidone
P.*aeruginosa*: *Pseudomonas aeruginosa*
PNIPAAm: Poly(N-isopropylacrylamide)
SA: Sodium alginate
S. *aureus*: *Staphylococcus aureus*
S. *epidermidis*: *Staphylococcus epidermidis*
SECs: Sunflower pollen grain exine capsules
SF: Silk fibroin
SFCH: Silk fibroin composite hydrogel
sEVs: Small extracellular vesicles
SLNs: Solid lipid nanoparticles
TA: Tannic acid
